# Effect of Significant Postoperative Complications on Decision Regret After Laparoscopic Sleeve Gastrectomy: a Case–Control Study

**DOI:** 10.1007/s11695-022-06113-w

**Published:** 2022-05-27

**Authors:** Katarzyna Bartosiak, Michał R. Janik, Maciej Walędziak, Krzysztof Paśnik, Andrzej Kwiatkowski

**Affiliations:** 1 Department of General, Oncological, Metabolic and Thoracic Surgery, Military Institute of Medicine, 128 Szaserów St, 04-141 Warsaw, Poland; 2 Department of General Surgery, Military Institute of Aviation Medicine, 54/56 Krasińskiego St, 01-755 Warsaw, Poland; 3 Department of General, Gastroenterological and Oncological Surgery, Collegium Medicum of the Nicolaus Copernicus University, 53-59 St. Joseph St, 87-100, Toruń, Poland

**Keywords:** Laparoscopic sleeve gastrectomy, Regret score, Complications, Weight loss

## Abstract

**Background:**

Thus far, no data are available on decision regret about sleeve gastrectomy (SG), particularly in patients who experienced perioperative complications. This study aimed to assess whether patients with postoperative complications regret their decision to undergo laparoscopic SG more than patients with an uneventful postoperative course.

**Methods:**

The study group comprised patients with complications after laparoscopic SG (cases). The control group comprised patients who did not experience any postoperative complications (controls). A telephone survey was conducted on all patients. Patients’ satisfaction regarding their decision to undergo surgery was assessed using the Decision Regret Scale.

**Results:**

In total, 21 patients who experienced postoperative complications and 69 controls were included. The patients in the study and control groups achieved similar percentages of total weight loss (32.9 ± 11.9 vs. 33.8 ± 15.0, *p* = 0.717) and excessive body mass index loss (74.9 ± 30.7 vs. 73.1 ± 36.7, *p* = 0.398) at 1 year postoperatively. The difference in weight change at 12 months postoperatively was not significant in both groups. The mean regret scores in the study and control groups were 13.2 ± 1.2 (range, 28–63) and 13.3 ± 1.1 (range, 12–66) (*p* = 0.818), respectively. Moreover, no significant difference was found among patients who expressed regret between the study and control groups (regret score > 50; 4.76% vs. 4.35%) (*p* = 1.000).

**Conclusion:**

This study suggests that patients with postoperative complications do not regret their decision to undergo SG more than patients with an uneventful postoperative course.

**Graphical abstract:**

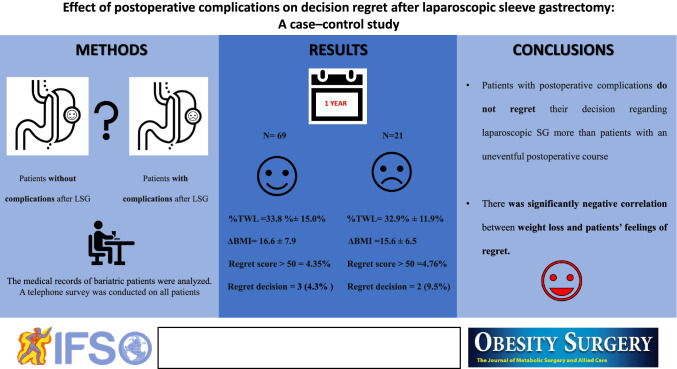

## Introduction

Bariatric surgery is considered the most effective treatment for morbid obesity and its associated comorbidities [1–4]. According to the American Society for Metabolic and Bariatric Surgery, the number of bariatric procedures performed annually has greatly increased in the last decade, with approximately 252,000 procedures performed in the USA in 2018 [5]. Recent studies have shown markedly improved safety and outcomes after bariatric surgery compared with those after other common general surgical procedures [6]. With this increasing demand for bariatric surgery, there are a growing number of patients who experienced postoperative complications.

Although complications after laparoscopic sleeve gastrectomy (SG) are rare [7, 8], they may negatively affect patient satisfaction. Several studies have assessed patients’ experiences with bariatric surgery with respect to their quality of life, weight loss, or comorbidity resolutions [9–12]. However, no data are available about decision regret regarding the surgery, particularly in patients who experienced perioperative complications.

This study aimed to assess whether patients with postoperative complications regret their decision to undergo laparoscopic SG more than patients with an uneventful postoperative course.

## Methods

### Study Design

After obtaining Institutional Review Board approval (10/WIM/2020), we retrospectively reviewed all bariatric surgery cases performed at our institution between 2016 and 2019. An electronic database was used to extract patient demographics, surgical history, complications, and postoperative outcomes. Patients with complications after laparoscopic SG were identified as cases and were included in the study group.

**Fig. 1:**
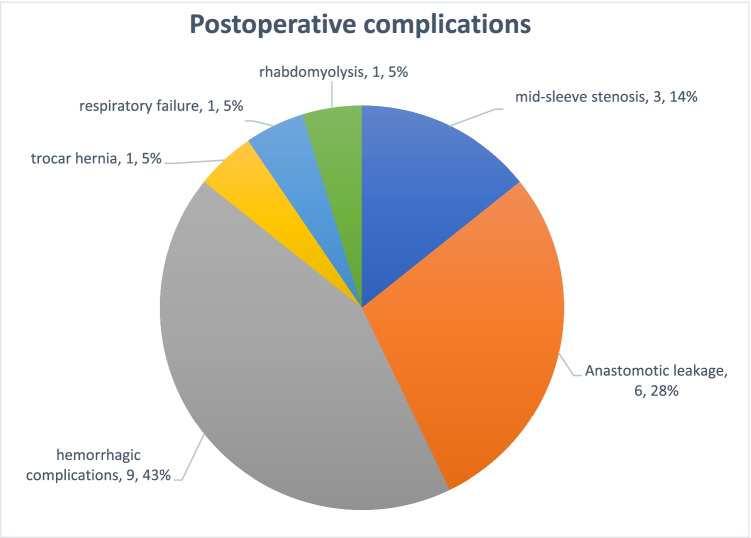
Postoperative complications, *n* (%)

A surgical complication was defined as any deviation from the ideal postoperative course that is not inherent in the procedure and does not include therapeutic failure [13]. Complications included anastomotic leakage requiring relaparotomy or endoscopic procedure, hemorrhagic events, anastomotic stenosis, lung failure requiring intubation, and rhabdomyolysis. All complications were graded 3 or 4 based on the Clavien–Dindo classification [13] (Fig. 1).

Subsequently, we randomly selected 100 patients who underwent laparoscopic SG during the same period and did not experience any postoperative complications and included them in the control group.

A telephone survey was conducted among all patients. If the patients were not contactable after 3 attempts during the week, contact details were considered outdated, and these patients were excluded from the study. Information regarding patient demographics, weight loss, comorbidities, and satisfaction with the surgery was collected.

### Instruments

Patient satisfaction regarding the decision to undergo SG was assessed using the Decision Regret Scale, which was developed by Brehaut et al. [14] to portray decision-making about hormone replacement therapy. A modified version of this scale was previously used for bariatric surgery [12, 15]. Patients were asked 5 questions (Table 1) to assess their feelings about undergoing surgery. Questions 2 and 4 were reverse coded. The scale ranged from 0 to 100, and higher scores reflected patients’ regret about undergoing surgery. A score of > 50 signified overall regret regarding their decision to undergo SG.

**Table 1:** Patients’ responses on LSG

	**Complications group *n* = 21 (%)**	**Control group *n* = 69 (%)**	***p* value**
Definitely made the right decision			*p* = 0.005
Definitely yes	12 (57.1%)	60 (86.9%)	
Probably yes	7 (33.3%)	5 (7.2%)	
Probably no	2 (9.5%)	2 (2.9%)	
Definitely no	0 (0%)	2 (2.9%)	
Regret choice			*p* < 0.001
Very much	1 (4.8%)	0 (0%)	
Somewhat	1 (4.8%)	3 (4.3%)	
A little	5 (23.8%)	1 (1.4%)	
Not at all	14 (66.7%)	54 (78.3%)	
Would do it again			*p* = 0.034
Very likely	14 (66.7%)	61 (88.4%)	
Somewhat likely	5 (23.8%)	2 (2.9%)	
Not very likely	1 (4.8%)	2 (2.9%)	
Not at all	1 (4.8%)	4 (5,8%)	
LSG caused negative effects			*p* = 0.011
A lot	1 (4.8%)	2 (2.9%)	
Some	3 (14.3%)	2 (2.9%)	
A little	3 (14.3%)	7 (10.1%)	
Not at all	14 (66.7%)	75 (84.1%)	
How wise was decision			*p* = 0.141
Very wise	17 (80.1%)	63 (91.3%)	
Somewhat wise	2 (9.5%)	3 (4.3%)	
Not very wise	1 (4.8%)	3 (4.3%)	
Not wise at all	1 (4.8%)	0 (0%)	

### Data Analysis

Analysis was performed using SAS software, University Edition (SAS Institute, Cary, NC, USA). Normality was checked using the Shapiro–Wilk test. Continuous variables were compared using the Mann–Whitney *U* test or unpaired Student’s *t*-test. Categorical variables were compared using the *χ*^2^ and Fisher’s exact tests. A correlation analysis was used to investigate the association between weight loss and total score on the Decision Regret Scale. Logistic regression was performed to assess the associations between age and body mass index (BMI) and regret. Weight loss was expressed according to the standards developed by Brethauer et al. [16] as a change in BMI (ΔBMI), percentage of total weight loss (%TWL), and percentage of excessive BMI loss (%EBMIL). Statistical significance was indicated by *p* < 0.05.

## Results

**Fig. 2:**
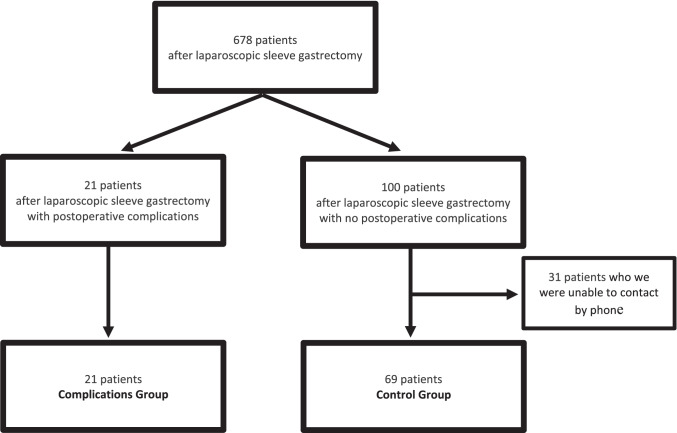
Flowchart of the inclusion of patients in the study

From 2016 to 2019, 678 patients underwent laparoscopic SG in our institution. Among them, 21 patients who experienced postoperative complications and 69 controls were included in this study. No patients refused to answer questions or participate in the study. However, we were unable to contact 31 patients (Fig. 2).

The mean ages of patients in the study and control groups were 46.5 (range, 28–63) and 44.1 (range, 22–66) years, respectively. Their mean preoperative BMIs were 47.4 ± 7.3 kg/m^2^ and 49.0 ± 7.4 kg/m^2^ (*p* = 0.476), respectively. Moreover, 33% of the patients in the study group and 45% in the control group were women. Comorbidities included hypertension [study group: *n* = 15 (71.4%); control group: *n* = 45 (65.2%); *p* = 0.792] and diabetes mellitus [study group: *n* = 9 (42.9%); control group: *n* = 34 (49.3%); *p* = 0.628]. Patients’ demographic characteristics are summarized in Table 2.

**Table 2:** Basic characteristics of the groups

Basic characteristic	Complications group	Control group	*p* value
Age, mean (years)	46.5 (± 9.9)	44.1 (± 10.8)	0.377
Sex			0.451
Female, *n* (%)	7 (33.3%)	31 (44.9%)	
Male, *n* (%)	14 (66.7%)	38 (55.1%)	
BMI, mean (kg/m^2^)	47.4 kg/m^2^ (± 7.3)	49.0 kg/m^2^ (± 7.4)	0.476
Smoker, *n* (%)	9 (42.9%)	22 (31.8%)	0.434
Comorbidities			
Diabetes mellitus, *n* (%)	9 (42.9%)	34 (49.3%)	0.628
Hypertension, *n* (%)	15 (71.4%)	45 (65.2%)	0.792
Obstructive sleep apnea, *n* (%)	7 (33.3%)	9 (13.0%)	0.049
Cardiovascular disease, *n* (%)	2 (10.0%)	7 (10.1%)	1.000
Hyperlipidemia, *n* (%)	10 (47.6%)	9 (13.0%)	0.002
Respiratory system diseases, *n* (%)	4 (19.1%)	1 (1.5%)	0.010

*BMI*, body mass index

The mean BMI at 12 months postoperatively was lower in the study group than in the control group (31.2 ± 6.1 vs. 32.4 ± 8.8 kg/m^2^, *p* = 0.312). ΔBMI at 12 months postoperatively tended to be lower in the study group than in the control group (15.6 ± 6.5 vs. 16.6 ± 7.9 kg/m^2^, *p* = 0.941). The patients in the study and control groups achieved similar %TWL (32.9 ± 11.9 vs. 33.8 ± 15.0, *p* = 0.717) and %EBMIL (74.9 ± 30.7 vs. 73.1 ± 36.7, *p* = 0.398) at 1 year postoperatively. The difference in weight change at 12 months postoperatively was not significant. Table 3 presents the weight loss outcomes of patients in the study and control groups.

**Table 3:** Weight loss outcome 12 months after sleeve gastrectomy

	Complications group	Control group	*p* value
BMI, mean (kg/m^2^)	31.2 ± 6.1	32.4 ± 8.8	*p* = 0.312
ΔBMI mean (kg/m^2^)	15.6 ± 6.5	16.6 ± 7.9	*p* = 0.941
%TWL	32.9 ± 11.9	33.8 ± 15.0	*p* = 0.717
%EBMIL	74.9 ± 30.7	73.1 ± 36.7	*p* = 0.398

*ΔBMI*, change in body mass index; *%EBMIL*, excess body mass index loss; *%TWL*, percent total weight loss

The postoperative hospital length of stay was significantly longer in the study group (19.7 ± 21.9 days) than in the control group (4.0 ± 0.9 days) (*p* < 0.001). The mean regret scores of patients in the study and control groups were 13.2 ± 1.2 (range, 28–63) and 13.3 ± 1.1 (range, 12–66) (*p* = 0.818), respectively. Age was negatively correlated with regret score (*r* =  − 0.103; *p* = 0.676), albeit not significantly. However, %TWL was significantly negatively correlated with regret score (*r* =  − 0.6.15; *p* = 0.005).

A score of > 50 on the Decision Regret Scale was considered to represent overall regret regarding the decision. Upon comparing the study and control groups, no significant difference was noted between patients who expressed regret (regret score > 50; 4.76% vs. 4.35%) (*p* = 1.000).

Table 1 shows detailed results of the regret score. Only 2 (2.9%) patients in the control group expressed dissatisfaction with their decision. Despite complications, 14 (66.7%) patients in the study group did not experience any negative effects of the surgery. When asked whether they regretted their decision, 2 (9.5%) patients in the study group and 3 (4.3%) patients in the control group answered positively (*p* < 0.001). Moreover, in the study group, 19 (90.5%) patients claimed that if they were given the choice again, they would be very likely or somewhat likely to make the same decision; similarly, 63 (91.3%) patients in the control group claimed that they would make the same decision again (*p* = 0.034).

## Discussion

With the increasing global prevalence of obesity, the number of patients requiring bariatric procedures continues to grow [17]. The efficacy of bariatric surgery, considering the improvements in the comorbidity course, daily functioning, and quality of life, has already been established [4, 18]. Despite the benefits of bariatric surgery, there remain serious concerns regarding postoperative complications.

Limited data are available on how patients feel about their decision to undergo SG in retrospect. This is especially true for patients who have experienced postoperative complications. As a continuation of a previous study [12], we wanted to assess whether patients with postoperative complications regret their decision to undergo SG more than patients with an uneventful postoperative course.

Our analysis showed comparable regret after SG in both groups of patients. We observed that the percentages of patients with a score of > 50 on the Decision Regret Scale, which is considered to represent overall regret regarding the patient’s decision, were almost the same in both groups.

This study strengthens the evidence [11, 12, 15] that weight loss is a factor that significantly affects patient satisfaction after bariatric surgery. Our findings highlighted that the regret associated with undergoing SG is correlated with worse weight loss outcomes than anticipated. Moreover, our study showed that the study and control groups achieved similar weight loss outcomes and regret scores.

To evaluate and provide recommendations on follow-up care for bariatric surgery, patients’ perspectives can provide valuable information [19]. Most previous studies on bariatric surgery primarily focused on clinical outcomes rather than patients’ experiences. Coulman et al. [11, 20] and Parretti et al. [21] recently demonstrated the long-term effects of bariatric surgery on various aspects of people’s lives and the challenges experienced by the patients after bariatric surgery. The results of our study confirmed the complexity of patients’ experiences. Surprisingly, patients with postoperative complications reported a similar degree of negative effects after SG compared with patients with an uneventful postoperative course.

The importance of a better understanding of factors that attenuate patient satisfaction was highlighted by Wallace et al. [22]. The study was designed to explore the predictors of post decision dissonance following bariatric surgery. They reported that of the 184 responders, 20 (10.9%) patients would not choose to undergo bariatric surgery if they had to do it over again. Their study shows the importance of preoperative counseling on managing postoperative expectations and outcomes is critical to a patient’s objective and subjective success.

The authors admit there is a need to analyze the causes of patients’ dissatisfaction after bariatric treatment more thoroughly. Although the purpose of this study does not take this analysis into account and only shows whether patients regret their decisions, it shows the importance of patients’ perspective on bariatric surgery. The intention of the researchers is to conduct further research exploring this issue.

Among bariatric surgery procedures, the overall complication rate is reported to vary from 4.3 to 14.5%, without significant differences between laparoscopic SG and laparoscopic gastric bypass procedures [23–25]. Although these numbers do not appear to be very high, the patients experiencing complications usually have different viewpoints. It has been proven that compared with medical specialists, patients perceive complications to have a greater effect on their health [26]. The findings of this study deepen our understanding of patients’ perspectives on the success or failure of bariatric procedures. Even patients who experienced perioperative events may not regret their decision to undergo surgery if the final outcome is satisfactory.

In 2006, Livingston et al. [27] were the first to identify smoking as an independent risk factor for worse outcomes after bariatric surgery. Since then, several studies have described the effects of smoking on postoperative outcomes. Many studies have directly examined the effects of smoking on specific complications after bariatric surgery and reported a strong association between smoking within 1 year before surgery and 30-day postoperative morbidity [28–31]. Our study appears to support these data. In this study, approximately 43% of the patients in the study group were smokers compared with 32% in the control group [9 (42.9%) vs. 22 (31.8%), respectively; *p* = 0.434].

Despite the lack of studies qualitatively describing patients’ feelings after bariatric surgeries, few studies have examined patient satisfaction after Roux-en-Y gastric bypass (RYGB). Turrentine et al. [32] reported that 99% of the participants rated their satisfaction with RYGB as high (mean score of 8.4, with a score of 10 representing high satisfaction). Wee et al. [15] quantified patients’ levels of regret after both RYGB and gastric banding and reported that up to 20% of patients who had undergone gastric banding expressed regret with having undergone the procedure, whereas approximately 4–8% of the patients who had undergone RYGB scored > 50 on the Decision Regret Scale (i.e., overall regret with their decision). Our data corroborate the research investigating regret after RYGB, consistent with a general trend whereby weight loss has the greatest effect on patients’ expressed regret after bariatric surgery.

The financial side of bariatric treatment is also worth emphasizing. In our institution, all operations, the entire hospital stays, and all costs are financed by National Health Fund, so patients do not pay for the surgery. Patients who experience complication are not exposed to higher costs for their recovery. As a result, patients experience and their opinion on the course of treatment are limited only to medical and personal issues.

## Limitations

This study has several limitations related to its retrospective design and small sample size. First, the limited sample size reduces the accuracy of the weight loss assessment. Second, the risk of selection bias is attributable to our inability to contact each patient who underwent SG. Third, the follow-up survey was performed via telephone, which may have been affected by recall bias. Finally, although all patients in the study group were followed up because of more detailed and frequent control visits, many patients in the control group were lost to follow-up because of our inability to contact patients owing to outdated phone numbers.

## Conclusion

The results of this study suggest that patients with postoperative complications do not regret their decision regarding laparoscopic SG more than patients with an uneventful postoperative course. Although further research is warranted, our findings highlight the importance of the patient’s viewpoint on the postoperative course and follow-up outcome evaluation.
